# How biofilm changes our understanding of cleaning and disinfection

**DOI:** 10.1186/s13756-023-01290-4

**Published:** 2023-09-07

**Authors:** Jean-Yves Maillard, Isabella Centeleghe

**Affiliations:** School of Pharmacy and Pharmaceutical Sciences, Cardiff University, Redwood Building, King Edward VII Avenue, Cardiff, CF10 3NB Wales, UK

**Keywords:** Biofilm, Dry surface biofilm, Disinfection, Resistance

## Abstract

Biofilms are ubiquitous in healthcare settings. By nature, biofilms are less susceptible to antimicrobials and are associated with healthcare-associated infections (HAI). Resistance of biofilm to antimicrobials is multifactorial with the presence of a matrix composed of extracellular polymeric substances and eDNA, being a major contributing factor. The usual multispecies composition of environmental biofilms can also impact on antimicrobial efficacy. In healthcare settings, two main types of biofilms are present: hydrated biofilms, for example, in drains and parts of some medical devices and equipment, and environmental dry biofilms (DSB) on surfaces and possibly in medical devices. Biofilms act as a reservoir for pathogens including multi-drug resistant organisms and their elimination requires different approaches. The control of hydrated (drain) biofilms should be informed by a reduction or elimination of microbial bioburden together with measuring biofilm regrowth time. The control of DSB should be measured by a combination of a reduction or elimination in microbial bioburden on surfaces together with a decrease in bacterial transfer post-intervention. Failure to control biofilms increases the risk for HAI, but biofilms are not solely responsible for disinfection failure or shortcoming. The limited number of standardised biofilm efficacy tests is a hindrance for end users and manufacturers, whilst in Europe there are no approved standard protocols. Education of stakeholders about biofilms and ad hoc efficacy tests, often academic in nature, is thus paramount, to achieve a better control of biofilms in healthcare settings.

## Background

The term biofilm was first used in 1975 from the visualisation of biofilms in a trickling wastewater filter and it described the microbial community that adheres to both abiotic and biotic surfaces [1]. Microbial biofilms are the most prevalent form of natural ecosystems [2, 3] and often composed of a complex microbial community embedded in an extracellular polymeric matrix (EPS) containing polysaccharides, proteins, lipids, enzymes, extracellular-DNA (eDNA) and water [4, 5]. Interactions between species impact on biofilm formation, biofilm evolutionary fitness, metabolic cooperation, and contribute to an increased in antibiotic resistance and biofilm susceptibility to disinfection [6–9]. Yet most studies on antimicrobial resistance in biofilms are based on the use of mono-species [10, 11].

Biofilms have been at the forefront of healthcare research for many years due to their association with chronic wounds, urinary catheter infections, pneumonia, and medical devices [5, 12, 13]. Dispersion and dissemination of pathogens from a biofilm, whether that be in a host from medical devices, or on a near-patient surface, pose a greater risk of infection [14].

It has been estimated that between 65 and 80% of all bacterial and chronic infections arise from biofilms [15]. Biofilms are also a leading cause of catheter-associated urinary tract infections (CAUTI), which has been estimated to cost $451 million dollars/year in the USA alone [16]. Globally, it is estimated that the prevalence of multidrug resistance in biofilms from HAI ranges from 17.9 to 100%, with species such as *Staphylococcus aureus*, *Pseudomonas aeruginosa* and *Acinetobacter baumannii* as common causative organisms [17]. These figures are likely to increase due to frequent use of indwelling medical devices and other implants [18].

Whilst “hydrated” biofilms have been the most studied in the literature, biofilms formed on dry environmental surfaces, which have only been described since 2012 [19], are widespread in the healthcare environment [20–22].

This review presents an overview of the importance of biofilms in the healthcare environment and their challenges to infection control and prevention regimens. This review does not intend to provide an assertive narrative of all the literature dealing with biofilms and disinfection but is using examples pertinent to both the use of disinfectants and antibiotics, and hydrated and dry biofilms.

For the purpose of this review, cleaning is defined as the removal of dirt from surfaces, whilst disinfection concerns the reduction of microorganisms on surfaces as a result mainly of a microbiocidal effect, combined or not with mechanical removal. Biocides are defined as “*a chemical substance, mixture, or microorganism intended to control any harmful organism in a way that is not purely physical or mechanical*” (https://www.hse.gov.uk/biocides/introduction.htm; 21/08/2023). The term antimicrobial refers to both biocides and chemotherapeutic antibiotics. Resistance is defined as surviving bacteria to disinfectant or a disinfection process, or to a clinical concentration of an antibiotic.

## Bacteria in hydrated biofilms are more resilient than planktonic ones to cleaning and disinfection

Hydrated bacterial biofilms are composed of bacterial cells embedded in a matrix of extracellular polymeric substances (EPS) which includes polysaccharides, proteins, lipids, extracellular enzymes, metal ions, and eDNA [5], the composition of which depends on the bacterial species forming the biofilms and environmental location.

### Biofilm resistance to disinfection a multifactorial event

**Fig. 1:**
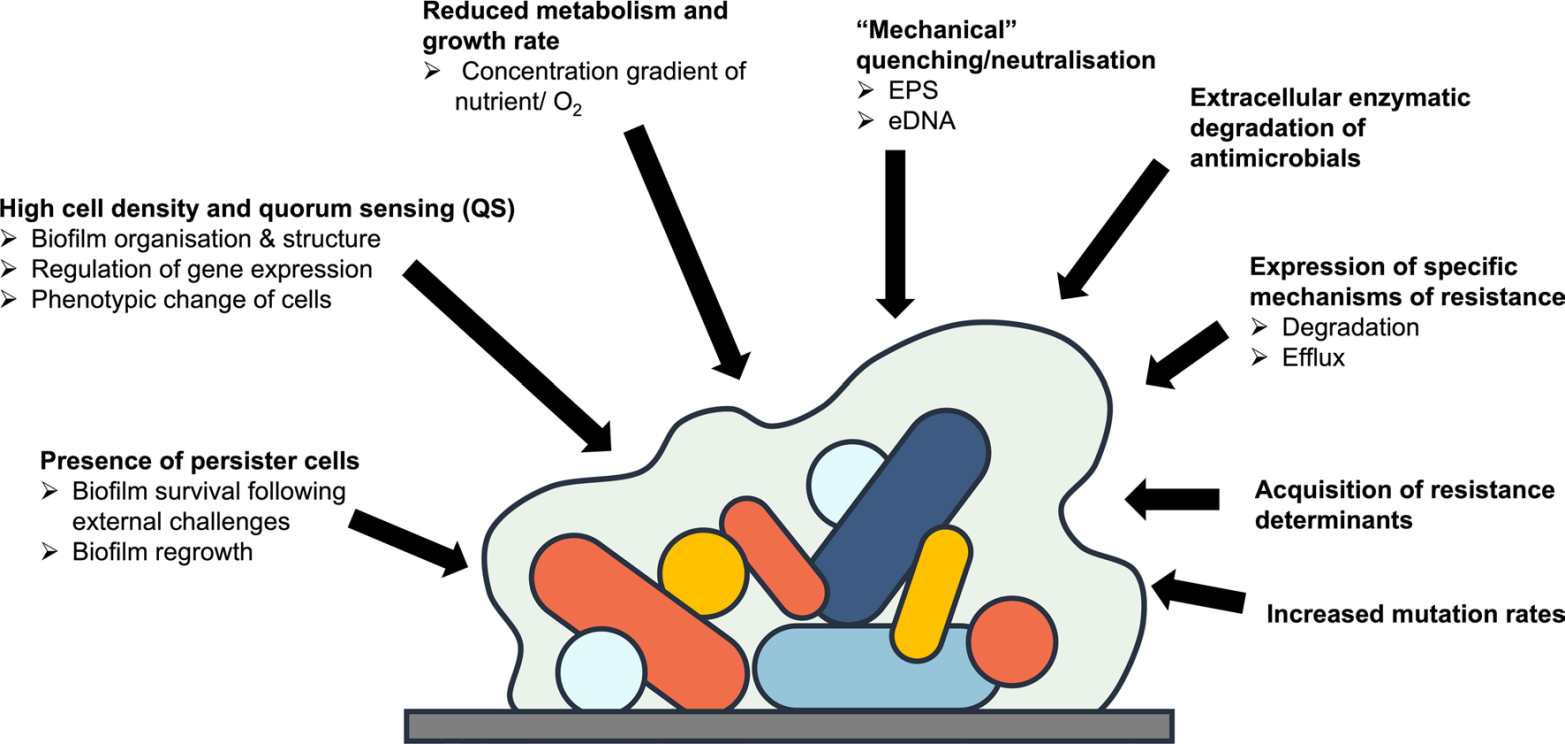
Mechanisms contributing to hydrated biofilm resistance to antimicrobials

Decreased susceptibility of bacteria embedded in hydrated biofilms to disinfection has been well reviewed over the years [23–26]. The reasons behind such a decrease in susceptibility is multifactorial (Fig. 1) [25–27] and include:

(i) “*Mechanical” quenching/neutralisation*: extracellular polymeric substances (EPS) matrix and to some extend lysed bacteria effectively act as organic load and contribute to the production of an antimicrobial concentration gradient [28]. eDNA in the EPS matrix contributes to antimicrobial resistance [29, 30] and horizontal resistance gene transfer [29]. (ii) *Reduced metabolism and growth rate*: the slow growth rate and metabolism of these bacteria lend themselves to the reduced efficacy of antibiotics in the treatment of biofilm infection, as many compounds rely on active metabolism to work [31, 32]. (iii) *High cell density and quorum sensing (QS)* plays an important role in biofilm formation and bacteria or biofilm raft detachment from biofilm [33, 34], but also has other functions including self-organisation and regulation of bacterial cells [35]. High cell density is required to have QS level impacting on cell signalling, gene expression and physiological changes in neighbouring cells [35–37]. (iv) *Presence of persister cells* which are metabolically inactive bacteria scattered through the biofilms, but different from the dormant bacterial cell population as a result, for example, of reduced access to nutrient or oxygen [38, 39]. It has been suggested that these cells are responsible for biofilm regrowth following biocide exposure [38, 39]. (v) *Extracellular enzymatic degradation of antimicrobials* [40]. (vi) *Expression of specific mechanisms of resistance* (e.g. degradation, efflux) in surviving bacteria, often located in “pockets” of survivors scattered through the biofilm. (vii) *Acquisition of resistance determinants* through increased horizontal gene transfer, including antimicrobial resistance genes (ARG) and quorum sensing genes [41, 42]. (viii) *Increased mutation rates* which can be associated with an increase in oxidative stress with a biofilm [43].

### The prominent role of EPS in biofilm resistance

EPS is a major contributor of biocide resistance of bacteria embedded in biofilms [44, 45], but not the sole contributor [46]. The EPS matrix is produced by microorganisms within the biofilm during the latter stages of biofilm development. EPS is considered as a defence barrier, but also it is an important factor in the development of new biofilms and biofilm dispersion. The dispersal of bacteria from biofilms is pertinent to healthcare since slough off biofilm parts can colonise new areas of either an environment or a host posing a severe threat [13]. Not only does dispersal play a role in the transmission of bacteria from biofilms, but patient areas are often crowded with equipment, lending themselves as a source for transmission. Any EPS which remains on an abiotic surface will also present a new structure for another biofilm to develop rapidly. The EPS matrix is also responsible for reduced nutrient and O_2_ in the depth of the biofilm, reducing metabolism and impacting growth rate. Bacterial cells residing in the depth of a biofilm have reduced metabolic activity due to the low oxygen concentrations [47].

### The impact of multispecies biofilm on resistance to disinfection

Multispecies biofilms are generally considered to be less susceptible than mono-species biofilms [9, 48, 49]. Some bacterial species within a complex biofilm have been shown to protect susceptible ones [10, 50, 51]. Bridier et al*.* [50] showed that *Bacillus subtilis* endoscope washer disinfector isolate, a strong EPS producer, which was shown to be resistant to chlorine dioxide (0.03%), hydrogen peroxide (7.5%) and peracetic acid (2.25%) [52] protected *S. aureus* from peracetic acid (0.35%) when in a biofilm. Likewise, *Acinetobacter johnsonii* was shown to protect *Salmonella enterica* subsp. *enterica* serovar Liverpool in a dual biofilm against benzalkonium chloride (300 mg/L) [53]. However, here, the decrease in susceptibility was associated with a change in outer membrane lipid composition driven by the presence of *A. johnsonii* [53]. Decreased biocide susceptibility to biocide following phenotypic bacterial adaptation within a biofilm has been described [54]. This is different from the impact of low concentrations of a biocide on bacterial phenotypic adaption within a biofilm, a phenomenon which has been well reported [25, 55] but not the subject of this review.

From the past literature, we know that high cell density on biofilm community structure plays an important role in biocide resistance [56–58]. Quorum sensing is a driving force for biofilm development, self-organisation and cell cooperation [35], but it also plays a role in other functions including, but not limited to, EPS synthesis, expression of virulence factors, antimicrobial including biosurfactant synthesis, extracellular enzyme synthesis [33, 59–63]. A critical concentration of QS-molecules needs to be reached to elicit a physiological response [37]. In biofilms QS molecules expression or accumulation is driven by high cell density [35–37, 64].

### Impact of biofilms on disinfectant efficacy

Bacteria embedded in biofilms are less susceptible than their planktonic counterparts [25, 39, 48, 49, 65–70] and might account for the failure of surface disinfection, with bacteria remaining on surfaces after biocide exposure [71, 72]. In addition, one needs to consider the impact of biofilm maturity. Gene expression controlling various metabolic activity and resistance mechanisms has been shown to change with biofilm aging [73]. Detached bacteria released from a biofilm present an intermediate susceptibility profile to biocides, somewhat between sessile and planktonic cell susceptibility [66, 69]. The resistance profile of detached bacteria can be somewhat related to the presence of EPS [44].

There have been many studies investigating the susceptibility of biofilms to disinfection. These studies highlighted that the lack of disinfectant efficacy was associated with biofilm thickness and maturity [66, 74, 75] or the presence of persisters [76] (Fig. 1). Whilst there are several parameters affecting biocide efficacy against biofilms, the use of quorum-sensing (QS) inhibitors has been explored with some success to potentiate antimicrobial efficacy [77, 78]. In addition, the surface material that harbours biofilms and the type of soiling (organic load) can impact on the ability of biocides or cleaning agents to remove biofilms [79].

## The impact of dry environmental surface biofilms on disinfection: a new paradigm

Environmental dry surface biofilms consist of multi species communities present on dry surfaces, embedded in EPS, and subjected to repeated desiccation periods. There are no official definitions of DSB yet, but DSB are not planktonic bacteria dried on surfaces. DSB were first reported in 2012 [19], whilst the term environmental dry surface biofilms was coined in 2015 [20]. DSB have been shown to be widespread in the healthcare environment [19–21, 80], with 90% of surfaces sampled [20] or more [21] potentially harbouring a DSB.

The two main in vitro protocols used to study DSB susceptibility to physical and chemical disinfection are based on a succession of hydrated and dry phase of an inoculum deposited on surfaces, either using the CDC biofilm reactor [81] or sedimentation biofilm [82]. Based on these methods, DSB have been shown to be less susceptible to physical and chemical disinfection/sterilisation. For example, the recovery of viable and culturable *S. aureus* from DSB after moist heat disinfection at 121 °C for 30 min was remarkable and differed from hydrated biofilms for which no bacteria were culturable; dry heat sterilisation at 121 °C for 20 min produced < 2 log_10_ reduction in viability in *S. aureus* DSB [83]. The efficacy of liquid disinfection against DSB depends overall on mechanical removal, formulation, but also soiling. Ledwoch et al. [84] showed that different biocides, including benzalkonium chloride (< 0.5%), peracetic acid (250 ppm), NaDCC (1000 ppm), NaOCl (1000 ppm), in combination with wiping reduced effectively (> 4 log_10_ reduction) a *S. aureus* DSB. However, when bacterial transfer from DSB post-disinfection wiping was assessed, only a couple of commercially available products prevented bacterial transfer (direct transfer or transfer via gloves) [84]. Although wiping on its own can remove *S. aureus* DSB from surfaces [85], Parvin et al. [86] showed that only a 1.4 log_10_ reduction in *S. aureus* DSB from surfaces could be achieved following 50 wiping actions using a standardised wiping process. In contrast, only 1 wiping action was sufficient to obtain a 3 log_10_ reduction of planktonic *S. aureus* dried on surfaces [86]. In the absence of mechanical removal, DSB can be very resilient to disinfection. Using live/dead staining, Almatroudi et al. [87] demonstrated that some *S. aureus* in DSB survive exposure to 20,000 ppm chlorine which produced a > 7 log_10_ reduction in viability and a reduced biofilm biomass by > 95%. Still viable *S. aureus* were able to regrow. *S. aureus* DSB exposure (5 min) to formulated peracetic acid (Proxitane), or chlorine (Chlorclean) were not efficacious producing < 3 log_10_ reduction in viability in the absence of soiling [88]. With soiling, all activity was lost. However, another peracetic acid formulation (Surfex) was shown to produce a > 6 log_10_ reduction in viability in the presence of organic load. Hydrogen peroxide (Oxivir) had no activity against *S. aureus* DSB [86]. Using *Bacillus licheniformis* DSB, Centeleghe et al. [89] showed an average of 2 log_10_ reduction from surfaces from a range of disinfectant-wipes following 10 s wiping at 500 g pressure and 60 s post-wiping before neutralisation.

Whilst the multispecies complexity of hydrated biofilms has been reported to protect susceptible bacteria from disinfection [9, 10, 50], the only DSB study to date did not report a protective effect of a less susceptible environmental bacterium (*B. licheniformis*) to *S. aureus* when exposed to disinfectants [89].

The mechanisms of DSB resistance to disinfection has not yet been widely studied, but considering the nature of DSB, low metabolism, desiccation and the presence of EPS are likely to contribute to the biofilm resistance to disinfection. Hu et al. [20] showed environmental DSB with very thick exopolymeric substance (EPS). Likewise, *S. aureus* artificial DSB has been shown to be embedded in EPS [81, 82], although, based on scanning electron microscopy images, the amount of DSB produced using the same in vitro protocol depends on the bacterial species [82, 89, 90].

Overall, EPS produced from DSB may be less than that of hydrated biofilms. DSB are likely to have a thickness of only tens of micron; approx. 30 μm for *S. aureus* DSB and 24–47 μm for environmental DSB [81], which profoundly differ from hydrated biofilms. Even though, EPS plays an important role in protecting bacteria from desiccation [91, 92]. It has also been suggested EPS is a major DSB mechanim of resistance to disinfection [81]. Changes to the bacterial cell structure in *S. aureus* DSB, notably the thickness of the cell wall, has been associated with a decreased susceptibility to sodium hypochlorite [93].

## Biofilm from drain in healthcare settings, a perpetual issue that needs addressing.

Biofilms on hydrated interfaces, such as sinks and taps, remain a problem in healthcare facilities [26]. There are numerous opportunities within plumbing systems throughout hospital buildings for bacteria to proliferate and form biofilms [94]. A main issue with sink traps and U-bends are that they are constantly hydrated, often humid and well protected environments. The steady supply of nutrients, seldom related to hand washing practices, and bacteria from workers hands and the disposal of various fluids, contribute to the development of microbial communities containing pathogenic organisms [95, 96]. The prevalence of multidrug resistant organisms within sink systems has been well documented, with common hospital-acquired organisms such as carbapenem-resistant Enterobacteriaceae [97]. *P. aeruginosa* is amongst one of the most commonly associated organisms, where baseline rates of colonisation have been found greater than 40% in all sinks in an intensive care unit [98]. Kotay et al. [99] have also documented the growth of *E. coli* up the sink unit from the P-trap to the strainer in 7 days, leading to droplet dispersal of the pathogen around the sink area, causing HAI concerns. Short stagnation times in sinks have also been shown to provide an opportunity for biofilm development and stagnant water allows for dispersal of cells from the biofilm [100]. The splash zone of a sink also causes problems since surfaces and objects nearby can become contaminated with sink pathogens when the sink is used [101, 102].

Numerous studies have investigated the impact of antimicrobial substances on resistance of biofilms formed in drain systems. There are many protocols and products to decontaminate sinks and drainage systems, but biofilms appear to regrow reasonably quickly after treatment. Ledwoch et al. [95] recreated a complex drain biofilm in a laboratory setting using environmental samples taken from U-bends. After treatment (3 doses for 15 min each) with commonly used disinfectants such as sodium dichloroisocyanurate (NaDCC; 1000 ppm), sodium hypochlorite (NaOCl; 1,000 ppm) non-ionic surfactant (< 5%) results displayed < 2.5 log_10_ reduction in biofilm formed in the section of the drain model, corresponding to the trap. In addition, bacterial bioburden in treated biofilm regrew to the same level as the untreated biofilm within 4 days, but for the PAA treated sample. Similarly, Buchan et al. [103] used a hydrogen peroxide and NaOCl-based foam on environmental sink samples from ICU. As with Ledwoch et al. [95], the disinfectants reduced the bacterial load, but biofilms were able to regrow within 7 days and reverted to pre-treatment levels. Unlike the findings presented above, a concentrated acetic acid formulation (20%) reduced CPE found in ICU sinks not only to lower than detectable levels, but also reduced patient acquisition of the pathogen [104]. Drain disinfection in hospital, although important to manage, needs to be practical in terms of treatment duration and safety. The regular use of products might be counterproductive as it may select for pathogenic species. In vitro studies have shown that prolonged use of quaternary ammonium compounds (QACs), resulted in the enrichment of Gram-negative species within the drain biofilm [105].

## Biofilm and medical devices, a lesson from history

Medical devices and implants have changed the science of medicine but come with an increased infection risk from the placement of foreign objects inside the body [106]. Biofilms are implicated in a multitude of diseases including catheter-associated infections and surgical site infections (Table 1) [12]. Catheter-associated urinary tract infections (CAUTI) are the most common biofilm-led infection from medical devices; approximately 150 million people worldwide develop CAUTI every year [107].

**Table 1:** Most frequently associated biofilms with medical device/implant infection and their most common causative organisms

Biofilm/disease	Causative species	References
Catheter-associated urinary tract infection (CAUTI)	*Escherichia coli* most common including resistant strains, *Enterobacter cloacae* heavy biofilm producer, *Klebsiella pneumoniae*	[111, 112]
Central line-associated bloodstream infection	Gram-positive organisms (coagulase negative Staphylococci, Enterococci, *Staphylococcus aureus*), *Candida albicans*	[113]
Surgical site infection (SSI)	*S. aureus*, coagulase-negative Staphylococci, *Enterococcus*, *E. coli*	[5]

As an implant or device enters the body, a film containing proteins will be produced around the object allowing for bacterial colonisation [108]. Once bacterial attachment has taken place, biofilms will start to develop. Upon reaching maturity, biofilm raft or bacteria will start dispersing, some entering the bloodstream causing serious infection [109]. Infection will often occur a few months following surgery or implantation of a device, but may be recognized as long as 24 months after [110].

Biofilms residing on device and implant surfaces are difficult to treat effectively. Preventative measures are much more appropriate for preventing biofilm colonisation. Antimicrobial coatings and surfaces have been developed to prevent biofilm formation. Although proven somewhat effective in the laboratory, in the natural environment microbial interspecies interactions present an obstacle for effectiveness, design and evaluation of such coatings [114]. Devices such as ventilators and catheters have parts that are disposable and so do not require decontamination following patient use. Those parts that can be in contact with a patient are required to be cleaned and disinfected following hospital guidelines. In the UK, government guidelines request that healthcare workers should manually clean and disinfect using an approved wipe product or cloth and approved liquid product [115].

Biofilms commonly colonise endoscopes despite following disinfection guidelines [116, 117]. High-level disinfection is used for endoscope reprocessing, usually following a manual or/and enzymatic cleaning step. Infections from endoscopes arise either endogenously from the patient’s own gut microflora, or, exogenously from contaminated equipment [118]. The small channel diameters of endoscopes make cleaning difficult; biofilms easily form on endoscopes’ lumens after sequential hydration and dehydration phases [119]. In accordance with international guidelines, it is essential that endoscopes are dried in a sterile air-drying cabinet and flushed with sterile air [120]. Drying is used to mitigate the risk of biofilm formation, as bacteria proliferate in wet environments [119]. However, after decontamination processes bacteria can remain within the device. Pajkos et al. [121] used scanning electron microscopy to identify bacteria embedded in biopsy channels from endoscopes taken from hospitals. The images show bacteria present in biofilms on the inside of the channels, suggesting current cleaning procedures are inadequate [122]. Of note, the structure of the biofilm identified by imaging [122] is very similar to that of DSB [19, 21, 89].

Clinical studies have shown that it takes as little as 30–60 days of use for biofilms to build up on endoscopes, whilst high level disinfection effectiveness might be limited [123]. Residual biofilm of *P. aeruginosa*, a common organism associated with endoscope infection, has been found to survive treatment with 4000 ppm of peracetic acid, largely over the concentration used for standard practices [124]. Other studies have shown that peracetic acid was effective in biofilms removal; however, when the drying process after disinfection was missed, regrowth of biofilms occurred within 48 h [125]. Heavy biofilm producers (such as *B. subtilis*) in addition to other bacteria (*Micrococcus luteus* and streptococci) have been recovered from automated endoscope washer disinfectors using chlorine dioxide [52]. The vegetative form of the *B. subtilis* isolate was resistant to chlorine dioxide (0.03% for 60 min in the presence of organic load), but also to hydrogen peroxide (7.5% for 30 min) and peracetic acid (2.23% for 5 min) [52]. The ability of surviving bacteria from a high-level disinfection process to become resistant to unrelated disinfectants has previously been reported with *Mycobacterium chelonae* following the use of glutaraldehyde 2% [126]

## Impact of biofilms for infection prevention and control

**Fig. 2:**
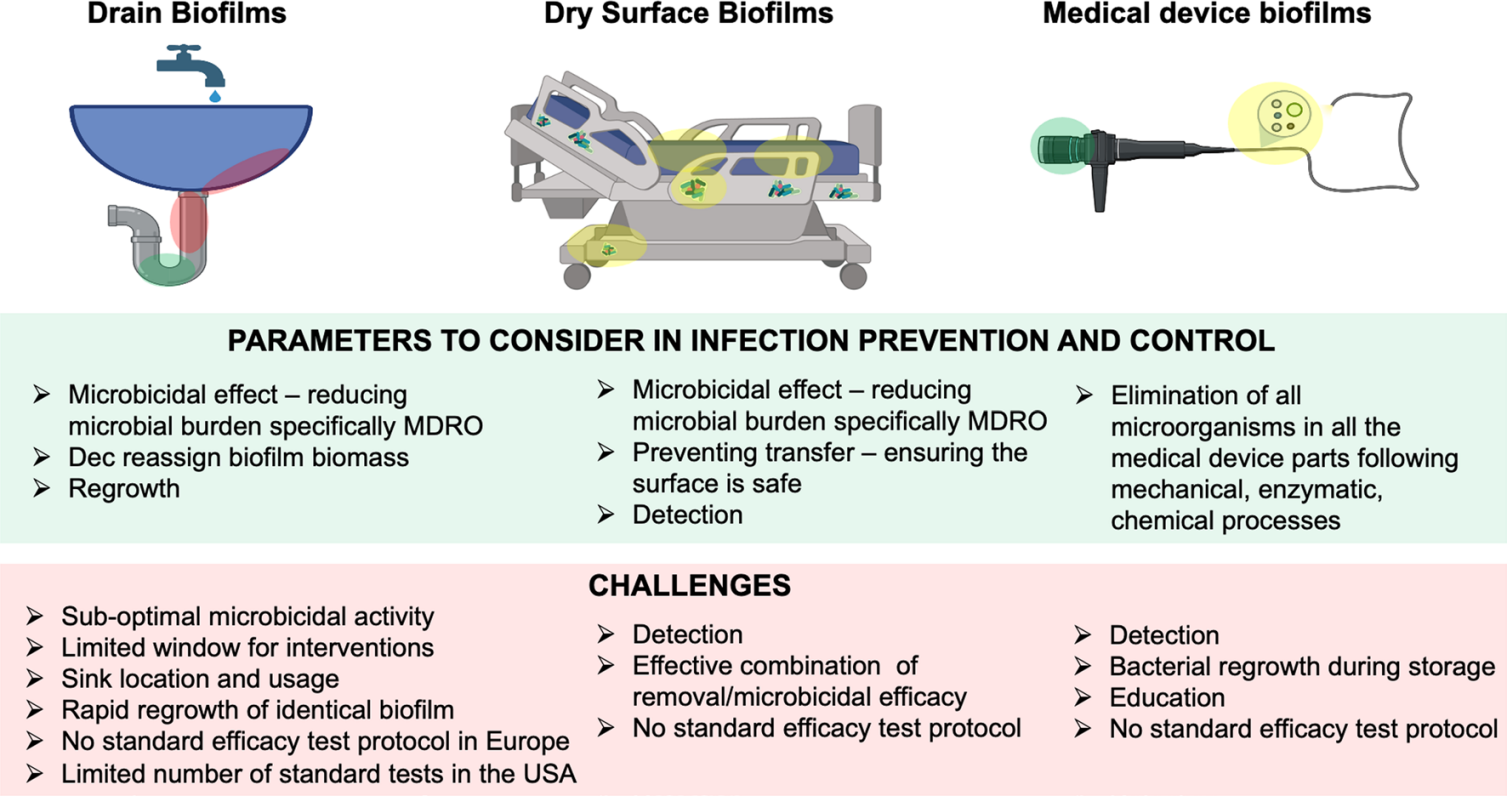
Biofilm type and location and associated challenges. Hydrated biofilms; dry surface biofilms; Semi-hydrated biofilm. Semi-hydrated biofilms are subjected to serial wet and dry phases, for example during device reprocessing

As presented above, biofilms are commonly found in the healthcare environment. They can harbour pathogens including multidrug resistant organisms (MDRO) and are responsible for HAI. Yet biofilms are difficult to control, and the use of cleaning and disinfection regimen is not always effective, particularly for medical devices, where single us items may be preferred. Where appropriate, the use of disinfectants is often sub-optimal against biofilms, resulting in high numbers of viable and culturable bacteria. Each biofilm type presents a specific challenge for disinfection (Fig. 2).

With drain biofilms, apart from the documented lack of disinfectant efficacy, the challenge is biofilm regrowth [95, 103]. Whilst some disinfectants such as chlorine-based ones can also decrease biofilm mass, rapid regrowth is inevitable, and the species composition of the biofilm may remain the same. Yet in vitro testing using a complex model has shown that the use of appropriate biocidal products may limit both surviving bacteria and regrowth [95]. Failing to control biofilm regrowth will lead to sink contamination and the potential spread of pathogens from water splashing. Physical measures to reduce splashing exist as well as sink design to prevent placing items on the sink ledge [127], whilst common sense would refrain to place sink < 2 m from patient’s bed or sterile preparation area.

The first description of DSB [19] led to the rapid consideration of their potential importance for microbial pathogens survival on dry environmental surfaces [128]. It is likely that DSB provides a mean for pathogen survival (including desiccation sensitive one) in a dry state in the environment, and act as a pathogen reservoir [26, 128]. In *S. aureus*, proteome analysis between DSB and hydrated biofilms showed differences in the up-regulation in DSB of proteins involved in peptidoglycan biosynthesis pathway related to cell-wall formation and thicker EPS matrix deposition, which was hypothesised to contribute to DSB persistence on dry surfaces [129]. Biofilms are likely to play a role in bacterial persistence, even Gram-negative ones, in dry environments. Espinal and colleagues [91] showed that biofilm-forming strains of *A. baumannii* survived better in surfaces than non-biofilm-forming ones. In addition, environmental studies describing the persistence of some pathogens, on surfaces in healthcare environment (reviewed in [26]) predate the first report of DSB [19].

The use of artificial DSB to measure the efficacy of cleaning and disinfection has provided useful information as to the resilience of microorganisms embedded in DSB to these processes [22, 82, 83, 85, 88–90, 130]. The successive hydrated and dry phases to form these in vitro DSB over a 12-day period [82, 87] reflects the succession of wet and dry phases provided from daily and terminal cleaning/disinfection in hospitals [26]. The efficacy of surface cleaning/disinfection against DSB has been recommended to be based on both reduction in viability from surfaces and decreased or lack of microbial transfer post-exposure [82, 84, 89, 90, 130], which principle originated from studies on pre-wetted antimicrobial wipes [131, 132] and is integral part of the ASTM2967-15 antimicrobial wipe standard efficacy test [133]. Bacterial transfer, including via the medium of gloves, is particularly relevant with DSB where the surface has been exposed to cleaning or disinfection [22, 90, 134, 135].

For medical devices, for which high-level disinfection is part of the reprocessing procedure, the presence of biofilms suggests that cleaning/disinfection protocols might be suboptimal [121]. Yet both hydrated biofilms and possible dry biofilms may be present and need to be effectively eliminated. Failure to do that might lead to bacterial growth during drying and storage of the device and increase the risk of HAI [119].

## Conclusions and further considerations

Biofilms are present in healthcare settings in the form of hydrated biofilms or DSB. With the exception to date of DSB—for which the information is not yet available—biofilms are associated with HAI. Yet the presence of DSB harbouring MDRO on surfaces is likely associated with persistence of pathogens in the environment and as such would impact HAI. There should be no doubt that biofilms need to be appropriately controlled although control measures may differ depending on the type of biofilms (Fig. 2). It is recommended for hydrated biofilm, such as drain ones, to investigate both a reduction in the microbial bioburden together with the time it takes for biofilm to regrow post-treatment [95]. This in essence will inform on the efficacy and duration of the treatment as well as how often the treatment needs to be applied. For DSB, mechanical removal together with disinfection have been shown to be efficacious, but information on transfer post-intervention is providing information on how safe the surface is. DSB can be disturbed following intervention and become transferable [22, 90, 134]. Although biofilms can contribute to the failure of infection prevention and control procedures, they may not solely be responsible for that since other factors are pertinent to the efficacy of disinfectant products [136]. Education of stakeholders, including infection control professionals is thus paramount to understand the risks associated with biofilms, and to apply appropriate mitigations to prevent contamination and infections.

## Data Availability

Not applicable.
